# Shotgun Metagenomic Sequencing of Gut Microbiota in Triplet Sibling with ASD and Gastrointestinal Symptoms: A Descriptive Case Report

**DOI:** 10.3390/children7120255

**Published:** 2020-11-25

**Authors:** Sabine Hazan, Kimberly D. Spradling-Reeves, Andreas Papoutsis, Stephen J. Walker

**Affiliations:** 1 Progenabiome™ Ventura Clinical Trials, 1835 Knoll Dr, Ventura, CA 93003, USA; drhazan@progenabiome.com (S.H.); papoutsis@progenabiome.com (A.P.); 2 Department of Internal Medicine, Wake Forest School of Medicine, Medical Center Blvd, Winston Salem, NC 27157, USA; kreeves@wakehealth.edu; 3 Wake Forest Institute for Regenerative Medicine, 391 Technology Way, Winston Salem, NC 27101, USA

**Keywords:** autism spectrum disorder, gastrointestinal, gut microbiome, metagenomic sequencing

## Abstract

The gut microbiome profile of a child with autism spectrum disorder (ASD) and co-occurring gastrointestinal (GI) symptoms was compared to that of her healthy triplet siblings to determine if she exhibited intestinal dysbiosis. Shotgun metagenomic sequencing was performed in individual fecal samples, and relative microbial abundance and diversity was determined. Microbial diversity was lower in sibling #3, coupled with a higher Bacteroidetes/Firmicutes ratio, a lower relative abundance of Actinobacteria, and an increased relative abundance of Proteobacteria. Our findings are suggestive of gut dysbiosis in a child with ASD and co-occurring GI symptoms, compared to her two healthy triplet siblings.

## 1. Introduction

Children with autism spectrum disorder (ASD) often experience chronic gastrointestinal (GI) symptoms (e.g., diarrhea, constipation, bloating, and/or gastroesophageal reflux (GERD)), and a strong positive correlation has been observed between the severity of GI symptoms and ASD severity [1]. Dysbiosis of the gut microbial composition has been documented in children with ASD [2,3,4,5], particularly in those with chronic GI symptoms, suggesting that gut microbiota play a critical role in behavioral impairment and GI symptoms in ASD and could serve as a novel target for therapeutic intervention.

Although several studies have reported significant differences in the gut microbiota profile in children with autism compared to non-autistic controls, a specific ASD ‘microbiome signature’ is still lacking due to the complex relationship of the microbiome with not only environmental factors and immune function, but also with the genetic background of the host [6]. In this brief report, we have characterized and compared the gut microbiota of triplet siblings (one male and two females, one of which had chronic GI symptoms and an ASD diagnosis) and their healthy mother to determine if perturbations in the microbiome are associated with ASD and/or co-occurring GI symptoms. The importance of a study like this is that, because the genetics and host environment were nearly identical for the three children, largely mitigating the genetic and environmental variability typically found in these of types of studies, this allows for exploratory comparison of whether the dysbiosis in sibling #3 may be associated with ASD, chronic GI symptoms, or both.

## 2. Materials and Methods

This study consisted of fecal microbiome analysis in a set of triplet children (age 7; two healthy children (one male and one female; siblings #1 and #2) and one female child (sibling #3) with chronic GI symptoms and ASD) Their healthy mother also underwent microbiome analysis for comparison.

Sibling #1 and #2 were healthy with no developmental delays, whereas sibling #3 was nonverbal and presented with a historical diagnosis of ASD and gastroesophageal reflux disease. The primary reported symptom was regurgitation. This was verified by physical examination and medical history, which was not consistent with the presence of more severe gastrointestinal disorders, such as celiac disease or Crohn’s disease. Sibling #3 had an Autism Treatment Evaluation Checklist (ATEC) score of 71, classifying her in the moderate to severe range. Sibling #3 was ruled out for celiac sprue, *Helicobacter pylori*, and inflammatory bowel disease (IBD), with an absence of fecal fat in the stools and with a fecal calprotectin < 15.6. Hemoccult testing was negative. Sibling #3 also had normal levels of C3, CH50, and was negative for Epstein-Barr virus (EBV) and Human polyomavirus 2 (JCV) and negative for presence of bacteria, parasites, or giardia in stool culture.

The triplet children all consumed essentially the same diet, consisting of a mix of western and south-central Asian foods, with no food aversion reported. At the time of this study, sibling #3 was taking lithium, as well as olive oil and fish oil supplements. Notably, lithium has been associated with increased microbiome species diversity and richness [7], which was not reflected in the decreased microbial diversity found in sibling #3. None of the children or the mother took antibiotics within the 3 months prior to sample collection. Of significance, all 3 children were delivered by C-section, were breast-fed for the first month, and then formula-fed thereafter, and all received the same vaccinations. Presentation of autism occurred in sibling #3 at 15 months.

Stool samples were collected using the OMNIgene^®^•GUT collection kit (DNAgenoTek™, Ottawa, ON, Canada), and microbial DNA was extracted from the samples using the QIAamp PowerFecal Pro DNA Kit (QIAGEN, Valencia, CA, USA). The DNA extracts were quantified using the QuantiFluor^®^ ONE dsDNA System and Quantus™ Fluorometer (Promega, Madison, WI, USA) and subsequently prepared for shotgun metagenomic sequencing using the Nextera™ DNA Flex Library Preparation Kit (Illumina, San Diego, CA, USA). The DNA libraries were normalized prior to pooling to ensure equal library representation, and the pooled libraries were sequenced using the Illumina NextSeq 550 System (Illumina, San Diego, CA, USA). Sequencing read depth ranged from 13.1 M to 15.4 M total reads in the four samples. Raw sequence reads (FASTQ files) were trimmed and analyzed using One Codex (https://www.onecodex.com/) to assess alpha diversity (as measured by the Shannon index) and to identify relative microbial abundances within the gut microbiome of each participant. Institutional Review Board (IRB) approval for human subject research was granted by the New England IRB #120190096 (Approval 8/26/2019). Written consent was obtained for each study participant.

## 3. Results

**Figure 1:**
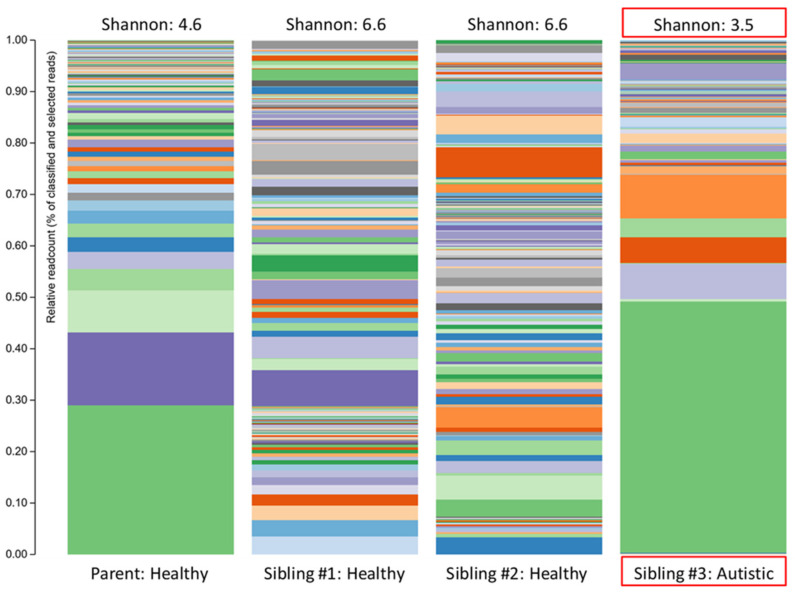
The stacked bar plots show the bacterial alpha diversity in the gut microbiome of a patient with autism spectrum disorder (Sibling #3) and the patient’s healthy biological triplet siblings and mother. Alpha diversity was quantified using the Shannon Index.

**Figure 2:**
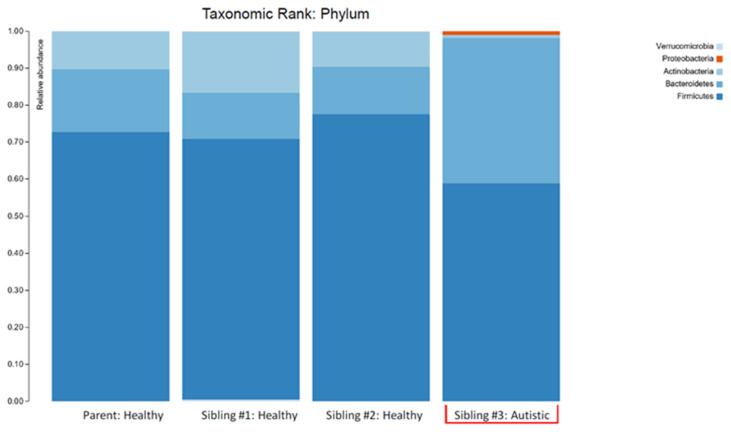
The stacked bar plots show the bacterial phyla composition in the gut microbiome of a patient with autism spectrum disorder (Sibling #3) and the patient’s healthy biological triplet siblings and mother.

The overall biodiversity of the gut microbiome was lower in triplet child #3 (Figure 1), and the relative abundance of specific phyla and genera varied when compared to the gut microbiome of the healthy siblings and mother. A higher *Bacteroidetes/Firmicutes* ratio and lower relative abundance of *Actinobacteria*, together with an increased relative abundance of *Proteobacteria*, were also observed in child #3 (Figure 2).

**Figure 3:**
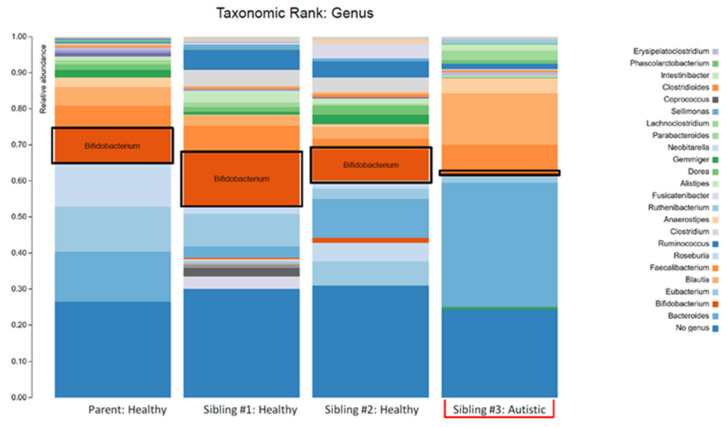
The stacked bar plots show the bacterial genera composition in the gut microbiome of a patient with autism spectrum disorder (Sibling #3) and the patient’s healthy biological triplet siblings and mother.

The higher *Bacteroidetes:Firmicutes* ratio in child #3 was attributed to a relative increase in *Bacteroidia*, particularly the *Bacteroides genera,* and a decrease in *Clostridia* (Figure 3; Table 1). The lower abundance of *Actinobacteria* in this sample was attributed to a decrease in the *Bifidobacterium genera* (Figure 3; Table 1). Despite the relative decrease of *Clostridia*, it should be noted that there was an increased abundance of *Blautia* in child #3 (Figure 3; Table 1).

## 4. Discussion

Gut dysbiosis has been identified in those with GI symptoms [8], autism [9], and GI symptoms and autism [10]; however, due to a number of complex and inter-related variables (e.g., microbiota diversity, host genetic background, host health status, etc.), we are not yet at the point where we have microbial ‘signatures’ that can unambiguously distinguish a healthy gut from an unhealthy gut. In this brief report, we describe the comparison of shotgun metagenomic sequencing data of stool samples from a set of triplet children (one with GI symptoms and ASD, the other two who were healthy) and their mother to determine if the sibling with autism and GI symptoms had a dysbiotic microbiome profile.

The plots in Figure 1 suggest: (i) the profile of the mother was different (less diverse) than siblings #1 and #2 and (ii) sibling #3 (with ASD and GI symptoms) had a profile that was markedly different from her two healthy siblings. The lower alpha diversity, higher *Bacteroidetes:Firmicutes* ratio, and findings of increased abundance of *Proteobacteria* and decreased abundance of *Actinobacteria* (especially the anti-inflammatory genera *Bifidobacterium*) in sibling #3 are in agreement with other studies that have compared the intestinal microbiota in ASD patients with healthy controls (reviewed in [5]) and are consistent with patterns reported in some ASD studies, while remaining non-specific in this single-case context. An alternative explanation is that the dysbiosis seen in sibling #3 may be related to GI symptoms, ASD status, or both, and these possibilities cannot be disentangled in a single-case observational report.

Aside from the obvious limitation that this report is based on findings from a single set of triplets, conclusions drawn from the results in this and other studies must be tempered by the understanding that this field is relatively new and, even in three recent systematic reviews of the literature, in all performing meta-analyses of published studies that evaluated gut microbiota in children with ASD [5,6,11], the authors reach differing conclusions: (i) “This systematic review demonstrated significant alterations of gut microbiota in ASD patients compared with HCs” [5]; (ii) “Research continues to report differences between microbiota of individuals with autism spectrum disorder and controls; however, the types and abundances of bacteria present remain inconsistent” [11]; and (iii) “results are inadequate to confirm a global microbiome change in children with ASD” [6]. While the degree and nature of the dysbiosis found in children with ASD are not currently agreed upon, a number of studies have reported dysbiosis as a feature observed in ASD when these children are compared to their neurotypical peers. Dysbiosis has also been reported in comparisons between siblings; however, this study is the first, to our knowledge, to compare triplets. As this study is exploratory and descriptive in nature, causal inferences or ASD-specific attributions are not supported by a single case with multiple uncontrolled confounders. Upper-GI symptoms, physiological factors, diet, stress, sleep, medications (including lithium), and supplements may also influence the gut microbiome and cannot be disentangled in this single-case observational report. The observed microbial features are consistent with some ASD reports but are non-specific and cannot be uniquely attributed to ASD in this context.

Perhaps in the case of this brief report, the details are less important than the bigger picture. A trio of young children, with nearly identical genetic and environmental backgrounds, were evaluated to determine if their microbiomes would reflect these similarities in make-up and exposures. The results showed concordance in two of the three children. The outlier had GI symptoms and autism. The dysbiosis that was evident in sibling #3 could be associated primarily with the GI symptoms, primarily with ASD, or with both GI symptoms and ASD. The potential elimination of two important confounding factors, environmental and genetic, makes this study the first of its kind, and thus noteworthy. Additional studies are needed to provide more clarity regarding these associations and to determine the direction of causality. Work is ongoing to define the role of the intestinal microbiota in ASD and to evaluate microbiome-related interventions more rigorously.

## Figures and Tables

**Table 1:** Comparison of the relative bacterial abundance in triplet siblings and their mother.

		Relative Abundance (%)			
		Mother	Sibling #1	Sibling #2	Sibling #3
		Healthy	Healthy	Healthy	ASD
Phylum	*Actinobacteria*	10.32	16.50	9.48	0.79
	*Bacteroidetes*	17.07	12.39	12.90	39.33
	*Firmicutes*	72.59	70.38	77.48	58.82
	*Bacteroidetes/Firmicutes ratio*	0.24	0.18	0.17	0.67
Genus	*Bacteroides*	13.84	3.14	10.76	34.57
	*Eubacterium*	12.47	9.00	2.79	1.86
	*Bifidobacterium*	9.88	15.44	8.70	0.70
	*Blautia*	5.33	2.68	3.38	14.30
Species	*Bacteroides uniformis*	<0.01	<0.01	<0.01	16.86
	*Bacteroides plebius*	11.72	0.00	1.01	17.71
	*Bacteroides vulgatus*	0.00	0.00	6.55	0.00
	*Roseburia faecis*	11.06	1.04	1.61	0.00
	*Bifidobacterium longum*	9.87	3.96	5.08	0.47
	*Bifidobacterium adolescentis*	0.00	11.48	3.62	<0.01
	*Clostridiales bacterium*	2.32	2.73	11.56	0.00
	*Escherichia coli*	0.00	0.00	0.00	0.62
