# Correction: Hazan et al. Shotgun Metagenomic Sequencing of Gut Microbiota in Triplet Sibling with ASD and Gastrointestinal Symptoms: A Descriptive Case Report. *Children* 2020, *7*, 255

**DOI:** 10.3390/children13070863

**Published:** 2026-06-29

**Authors:** Sabine Hazan, Kimberly D. Spradling-Reeves, Andreas Papoutsis, Stephen J. Walker

**Affiliations:** 1 Progenabiome™ Ventura Clinical Trials, 1835 Knoll Dr, Ventura, CA 93003, USA; drhazan@progenabiome.com (S.H.); papoutsis@progenabiome.com (A.P.); 2 Department of Internal Medicine, Wake Forest School of Medicine, Medical Center Blvd, Winston Salem, NC 27157, USA; kreeves@wakehealth.edu; 3 Wake Forest Institute for Regenerative Medicine, 391 Technology Way, Winston Salem, NC 27101, USA

## Title Updated

The title of this publication [1] has been updated to “Shotgun Metagenomic Sequencing of Gut Microbiota in Triplet Sibling with ASD and Gastrointestinal Symptoms: A Descriptive Case Report” to better reflect the descriptive nature of the study.

## Text Correction

There were some statements in the original publication that need clarification of interpretive language [1]. Following publication, the authors recognized that certain statements in the Introduction and Discussion could be read as implying a stronger degree of inference than warranted for a single-case exploratory observational report. The text has therefore been clarified to state more explicitly the descriptive nature of the study, the presence of uncontrolled confounders, and the non-specific character of the observed microbial features.

A correction has been made to Section 1, the second paragraph, the last sentence “The importance of a study like this is that, because the genetics and host environment were nearly identical for the three children, largely mitigating the genetic and environmental variability typically found in these of types of studies, this allows for the interpretation that the dysbiosis in sibling #3 is likely to be associated more directly with having either ASD, chronic GI symptoms, or both.” has been updated to: “The importance of a study like this is that, because the genetics and host environment were nearly identical for the three children, largely mitigating the genetic and environmental variability typically found in these of types of studies, this allows for exploratory comparison of whether the dysbiosis in sibling #3 may be associated with ASD, chronic GI symptoms, or both.”

A correction has been made to Section 2, the second paragraph, the third sentence “These was verified by physical examination and medical history, which was not consistent with the presence of more severe gastrointestinal disorders, such as celiac disease or Crohn’s disease.” has been updated to: “This was verified by physical examination and medical history, which was not consistent with the presence of more severe gastrointestinal disorders, such as celiac disease or Crohn’s disease.”.

A correction has been made to Section 4, the second paragraph “The plots in Figure 1 make two things clear: (i) the profile of the mother was different (less diverse) than siblings #1 and #2 and (ii) sibling #3 (with ASD and GI symptoms) had a profile that was markedly different from her two healthy siblings. The lower alpha diversity, higher *Bacteroidetes:Firmicutes* ratio, and findings of increased abundance of *Proteobacteria* and decreased abundance of *Actinobacteria* (especially the anti-inflammatory genera *Bifidobacterium*) in sibling #3 are in agreement with other studies that have compared the intestinal microbiota in ASD patients with healthy controls (reviewed in [5]) and therefore provide additional support for the concept of ASD-specific features of intestinal dysbiosis. An alternative explanation could be that the dysbiosis seen in sibling #3 is partially or largely due to her GI symptoms, but since the primary GI symptom was reflux (i.e., upper GI), it seems more likely that the dysbiosis is related to the ASD status, and therefore, the agreement with other findings in children with ASD is important.” has been updated to: “The plots in Figure 1 suggest: (i) the profile of the mother was different (less diverse) than siblings #1 and #2 and (ii) sibling #3 (with ASD and GI symptoms) had a profile that was markedly different from her two healthy siblings. The lower alpha diversity, higher *Bacteroidetes:Firmicutes* ratio, and findings of increased abundance of *Proteobacteria* and decreased abundance of *Actinobacteria* (especially the anti-inflammatory genera *Bifidobacterium*) in sibling #3 are in agreement with other studies that have compared the intestinal microbiota in ASD patients with healthy controls (reviewed in [5]) and are consistent with patterns reported in some ASD studies, while remaining non-specific in this single-case context. An alternative explanation is that the dysbiosis seen in sibling #3 may be related to GI symptoms, ASD status, or both, and these possibilities cannot be disentangled in a single-case observational report.”.

A correction has been made to Section 4, the third paragraph, the last two sentences “While the degree and nature of the dysbiosis found in children with ASD are not currently agreed upon, the field has come to accept that dysbiosis is a common feature of ASD when these children are compared to their neurotypical peers. Dysbiosis has also been reported in comparisons between siblings; however, this study is the first, to our knowledge, to compare triplets.” have been updated to: “While the degree and nature of the dysbiosis found in children with ASD are not currently agreed upon, a number of studies have reported dysbiosis as a feature observed in ASD when these children are compared to their neurotypical peers. Dysbiosis has also been reported in comparisons between siblings; however, this study is the first, to our knowledge, to compare triplets. As this study is exploratory and descriptive in nature, causal inferences or ASD-specific attributions are not supported by a single case with multiple uncontrolled confounders. Upper-GI symptoms, physiological factors, diet, stress, sleep, medications (including lithium), and supplements may also influence the gut microbiome and cannot be disentangled in this single-case observational report. The observed microbial features are consistent with some ASD reports but are non-specific and cannot be uniquely attributed to ASD in this context.”.

A correction has been made to Section 4, the fourth paragraph, the last sentence “Work is ongoing to define the role of the intestinal microbiota in ASD and how best to treat intestinal dysbiosis.” has been updated to: “Work is ongoing to define the role of the intestinal microbiota in ASD and to evaluate microbiome-related interventions more rigorously.”.

## Update to the Conflicts of Interest Statement

The conflicts of interest statement has been changed from “S.H. owns ProgenaBiome and Ventura Clinical Trials, which may constitute a competing interest. The other authors declare no conflicts of interest.” to “Sabine Hazan is the Founder and Chief Executive Officer of ProgenaBiome and Ventura Clinical Trials and holds patents and patent applications related to microbiome technologies. The other authors declare no competing interests.”.

These revisions clarify that the findings are descriptive and hypothesis-generating and do not support causal or condition-specific associations. This correction was approved by the Academic Editor. The original publication has also been updated.

## References

[1] Hazan S., Spradling-Reeves K.D., Papoutsis A., Walker S.J. (2020). Shotgun Metagenomic Sequencing of Gut Microbiota in Triplet Sibling with ASD and Gastrointestinal Symptoms: A Descriptive Case Report. Children.

